# Glass Fall-Offs Detection for Glass Insulated Terminals via a Coarse-to-Fine Machine-Learning Framework

**DOI:** 10.3390/mi17010128

**Published:** 2026-01-19

**Authors:** Weibo Li, Bingxun Zeng, Weibin Li, Nian Cai, Yinghong Zhou, Shuai Zhou, Hao Xia

**Affiliations:** 1 School of Information Engineering, Guangdong University of Technology, Guangzhou 510006, China; liweibo@shoulide.com (W.L.); zengbingxun1@mails.gdut.edu.cn (B.Z.); 3122002013@mail2.gdut.edu.cn (W.L.); zhouyh@gdut.edu.cn (Y.Z.); 2 China Electronic Product Reliability and Environmental Testing Research Institute, Guangzhou 510006, China; zhoushuai@ceprei.com (S.Z.); xiahao@ceprei.com (H.X.)

**Keywords:** glass insulated terminal, defect detection, coarse-to-fine machine learning, sector feature, GBDT

## Abstract

Glass-insulated terminals (GITs) are widely used in high-reliability microelectronic systems, where glass fall-offs in the sealing region may seriously degrade the reliability of the microelectronic component and further degrade the device reliability. Automatic inspection of such defects is challenging due to strong light reflection, irregular defect appearances, and limited defective samples. To address these issues, a coarse-to-fine machine-learning framework is proposed for glass fall-off detection in GIT images. By exploiting the circular-ring geometric prior of GITs, an adaptive sector partition scheme is introduced to divide the region of interest into sectors. Four categories of sector features, including color statistics, gray-level variations, reflective properties, and gradient distributions, are designed for coarse classification using a gradient boosting decision tree (GBDT). Furthermore, a sector neighbor (SN) feature vector is constructed from adjacent sectors to enhance fine classification. Experiments on real industrial GIT images show that the proposed method outperforms several representative inspection approaches, achieving an average IoU of 96.85%, an F1-score of 0.984, a pixel-level false alarm rate of 0.55%, and a pixel-level missed alarm rate of 35.62% at a practical inspection speed of 32.18 s per image.

## 1. Introduction

Sealing for the electronic components is an important protection method in the field of electronic packaging, since it can protect the electronic component from the interference, such as vibration and corrosion [1]. Glass is widely used as the sealing material for the electronic component, due to its advantages of excellent insulation, resistance to high temperature, high pressure, and chemical erosion [2,3]. When the metal is sealed with the glass in the high-temperature environment to compose a glass-insulated terminal (GIT), interfacial regions between the sealing glass and the metal form a strong chemical bond [4,5]. Thus, the GIT has sufficient tensile and torsional strength, which is suitable and widely used in oil, natural gas applications, high-temperature sensors, nuclear reactors, implantable medical devices, and batteries [6,7,8].

During the GIT manufacturing, the residual stresses generated during the cooling process of the sealing can decrease the sealing strength, which will induce the breakage of the sealing to cause the defects of fall-offs in the sealing glass of the GIT [9,10,11]. If this defective GIT works in a humid environment or for a long time, the metal in the GIT will migrate along the fall-offs, resulting in a short circuit [12]. In real industries, the surfaces of GITs are manually inspected by the quality control (QC) workers via human vision. Since the glass has the characteristics of the reflection of light, a long-term and high-intensity inspection will easily lead to the fatigue of the workers, which will further result in mis-inspection and even missed inspection [13,14,15]. Therefore, an automatic inspection is significant for the GIT manufacturer to alleviate the labor burden of the QC workers, and even to improve the yield of GITs.

Currently, automatic optical inspection (AOI) is widely applied as an effective nondestructive automatic inspection approach in electronic industry, and a large number of related AOI algorithms have been designed for surface defect detection [16,17,18,19]. However, most of existing AOI algorithms cannot be employed to inspect the glass fall-offs that emerge on the surfaces of GITs due to the complex characteristics of the reflection of light for the glass. Although, to the best of our knowledge, no reports study the inspection of glass fall-offs in GITs, some similar studies have been presented to inspect glass-packaged electronic components, ceramic devices, and metal–glass hybrid structures, where strong light reflection and non-uniform illumination often obscure defect boundaries and reduce the reliability of global image evaluation methods [12,13,14,15]. However, these similar studies cannot be directly transferred to inspect the glass fall-offs in GITs due to different defect characteristics. Only a few studies have focused on surface defect inspection rather than glass-offs for GITs [14,20,21]. Liu et al. combined traditional thresholding and morphological processing to locate stomatal bubbles in GITs used in aerospace equipment [14]. However, compared with stomatal bubbles, glass fall-offs usually demonstrate extremely irregular shapes. Due to the excellent self-learning ability of deep learning, they modified the Faster R-CNN to inspect GITs [21]. To deal with the insufficiency of the data for deep learning, a DCGAN was employed to generate a large number of simulated GIT images involving tiny stomatal bubbles on the GIT surfaces [20]. However, the glass fall-off regions in the GIT images demonstrate various appearances in shape, regularity, and color, which are more complicated than stomatal bubbles. Moreover, it is difficult to acquire a large number of GIT images with various fall-offs in the industry. Thus, the generated GIT images with glass fall-offs are most probably different from the real ones.

Although deep learning has been widely used for industrial visual inspection, some challenges emerge in real industries. For example, many manufacturers request that the AOI system should be deployed in a short period, typically within 60 days, which results in lack of available samples for training a promising deep-learning model. Meanwhile, inherent limitations—including scarce defect samples, exorbitant annotation costs, semantic deficiency in industrial images, and pronounced batch variations—severely constrain the applicability of data-driven methodologies. Consequently, the development of robust detection algorithms that synergistically integrate domain expertise and prior-engineered features while maintaining reliable performance under minimal sample conditions is also a pivotal breakthrough for industrial deployment.

To automatically inspect the glass fall-offs on the GIT surfaces, a coarse-to-fine machine learning is proposed based on the prior knowledge of GIT images, which subsequently involves the stages of image pre-processing, coarse classification, and fine classification. In the image pre-processing stage, an adaptive sector partition scheme is designed to partition the GIT image along the longitude and latitude sides. A new metric is proposed to comprehensively determine the appropriate number of the sectors, which together involves pixel-level evaluation, sector-level evaluation, and inspection time. In the coarse classification stage, four categories of sector features are designed to characterize each sector as the input of the gradient boosting decision tree (GBDT) [22], involving statistical color features, gray variations, reflective features, and statistical gradient directions. In the fine classification stage, a sector neighbor (SN) feature vector for each sector is designed based on the coarse classifications of its neighbor sectors, which can reveal its local neighbor characteristics. Finally, the four categories of sector features and the SN feature vector of the sector are input into the GBDT to distinguish whether the sector is defective.

The contributions of our work can be summarized as follows.

(1) To the best of our knowledge, we make the first attempt to automatically inspect glass fall-offs of GITs, which is implemented by a designed coarse-to-fine machine-learning framework involving adaptive sector partition, four categories of sector features, and sector neighbor features.
(2) An adaptive sector partition scheme is designed to partition the GIT image into some sectors of various sizes. To comprehensively determine the appropriate number of the sectors, a new metric is proposed by combining pixel-level evaluation and sector-level evaluation with inspection time.
(3) Four categories of sector features are designed to reveal the appearance characteristics of each sector. Specifically, a white ratio and a Canny coefficient are defined to compose the designed reflective features of the sector.
(4) A SN feature vector is designed to reveal the local neighbor characteristics of each sector, composed of coarse classifications of its neighbor sectors.

It should be noted that the experimental evaluation in this study is conducted on a relatively small industrial dataset, consisting of 43 GIT samples, among which 40 contain glass fall-off defects. Although this dataset reflects realistic manufacturing conditions where defective samples are scarce, the limited sample size may still constrain the statistical reliability and generalization of the reported results. To partially mitigate this issue, multiple randomized train–test splits, an independent test dataset, and the variance of IoU are reported to assess performance stability. Nevertheless, the findings should be interpreted with the understanding that further validation on larger and more diverse datasets is required to fully confirm the general applicability of the proposed framework.

## 2. Proposed Coarse-to-Fine Framework

### 2.1. Architecture of the Proposed Framework

**Figure 1:**
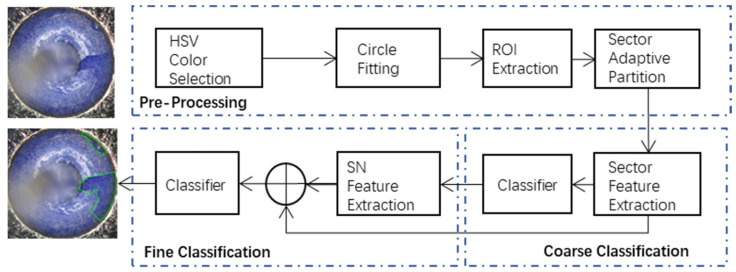
The architecture of the proposed coarse-to-fine framework. The framework is composed of pre-processing, coarse classification, and fine classification. The green regions in the example images indicate the detected glass fall-off areas produced by the proposed framework.

Figure 1 illustrates the architecture of the proposed coarse-to-fine framework for glass fall-off detection, which consists of three main stages: image pre-processing, coarse classification, and fine classification. In the image pre-processing stage, the acquired GIT image is successively processed by the operations of HSV color selection, circle fitting, and the region of interest (ROI) extraction. Then, an adaptive sector partition scheme is proposed to divide the extracted ROI into multiple sectors along the longitude and latitude directions, which fully exploits the geometric shape prior of the GIT. In the coarse classification stage, four categories of sector features are designed to characterize each sector and are subsequently input into a gradient boosting decision tree (GBDT) for coarse classification. To further capture local spatial consistency, a sector neighbor (SN) feature vector is constructed from the coarse classification results of neighboring sectors, thereby revealing local neighborhood characteristics. Finally, fine classification is performed by combining the sector features and the SN feature vector, which are jointly input into the GBDT to determine whether each sector contains glass fall-offs.

### 2.2. Image Preprocessing

**Figure 2:**
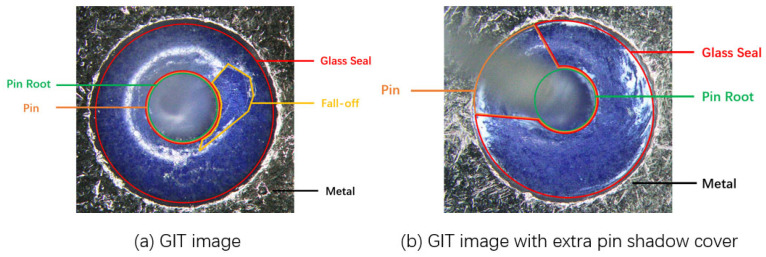
Two examples of GIT images. Each GIT involves sealing glass, a metal shell, and a pin.

Figure 2 illustrates two examples of GIT images acquired from two shooting angles, in which the ring-like glass is sealed between the metal shell and the pin. As illustrated in Figure 2, the acquired GIT image involves the regions of the pin, the sealing glass, and the peripheral metal. The pins located in the centers of the GITs demonstrate blurred and different shapes due to the shooting angle. The regions of the sealing glass demonstrate a blue-like characteristic due to the materials encapsulated in this kind of GIT. This blue-like characteristic is significant for extracting the circular-ring-like ROI from the GIT image. The ROI is possibly not an entire circular ring due to the shooting angle. As indicated in Figure 2a, a glass fall-off emerges in the right of the GIT. The fall-off region also demonstrates a blue-like characteristic in the GIT image, which will puzzle the inspection if only the color characteristics are considered. Thus, we design an image preprocessing scheme to extract the ROI of the GIT image for subsequent feature extraction, described as follows.

First, the GIT image is converted from the RGB space into the HSV space. Since the ROI of the GIT image is demonstrated in blue, the blue pixels are selected to form a rough mask of the GIT image, whose Hue values cover from 200 to 248, Saturation values from 43 to 255, and Value values from 46 to 255. Next, the rough mask is processed by the morphological operations of corrosion and expansion to achieve a circle mask. Subsequently, a simplified geometrical circle fitting method [23] is employed to achieve a ring mask, which determines the external and internal peripheries of the sealing glass of the GIT. Thus, the circular-ring-like ROI of the GIT image is extracted by the ring mask. Figure illustrates the processes of ROI extraction for a GIT image.

Due to the characteristics of the reflection of light for the glass, the ROI of the GIT image illustrates complicated color textures, which indicates that global evaluation cannot be suitable in this case. Due to the circular-ring-like shape for the ROI, an adaptive sector partition scheme is designed to divide the ROI into some sectors for local evaluation.

**Figure 3:**
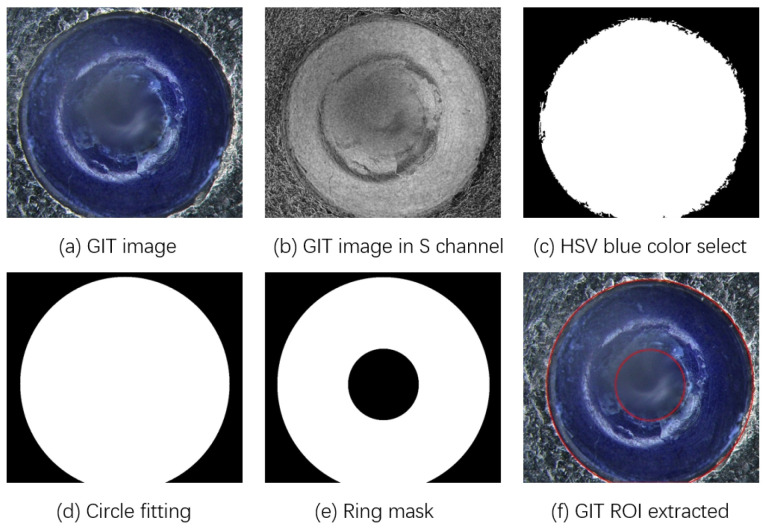
ROI extraction for a GIT image. (**a**) Acquired GIT image; (**b**) GIT image in S channel; (**c**) Rough mask via the blue-like pixel selection; (**d**) Circle mask via morphological operations; (**e**) Ring mask via the geometrical circle fitting method [23]; (**f**) ROI of the GIT image.

Considering the symmetrical characteristics of the circular ring, the ROI is partitioned into $2^{m+2}\;(m=1,2,\ldots ,M)$ sector-like sub-ROIs in the longitude side. To adapt to different types of GITs, a latitude partition coefficient c is defined as

(1) $$c=\left\lfloor \frac{R}{r}\right\rfloor$$

where *R* and *r* are the radii of the external and internal peripheries of the sealing glass, respectively. $\left\lfloor \cdot \right\rfloor$ is a rounding-up operation. c is fixed if the type of the GIT is given. In practice, based on statistical observation of the acquired GIT images, the ratio between the outer radius and the inner radius of the sealing glass region is approximately 3:1. Therefore, the latitude partition coefficient is empirically set to c = 3, which allows the radial partition to reasonably match the geometric structure of the sealing glass while maintaining balanced sector sizes. Then, the region involving the sealing glass and the pin root (as illustrated in Figure 3) can be partitioned into c(n + 1) (n = 1, 2, …, N) concentric circles. It is noted that the most internal (n + 1) concentric circles correspond to the region of the pin root.

Thus, the ROI of the GIT can be adaptively partitioned along the longitude and latitude directions into $(c-1)(n+1)\times 2^{m+2}$ sectors. And the number of sectors is determined by the two partition parameters m and n, which will influence the inspection. Specifically, with the increase of the two partition parameters m and n, the number of sectors increases, resulting in the increase of inspection time and the decrease of the size of each sector. Large sectors are beneficial for image global feature extraction but easy to submerge subtle details, and vice versa. Thus, appropriate partition parameters can provide appropriate sizes of the sectors, which will be experimentally discussed in Section 4.

### 2.3. Coarse Classification

To comprehensively characterize the sectors, four categories of sector features are designed for each sector, such as statistical color features, gray variations, reflective features, and statistical gradient directions. Then, the sector features are input into a GBDT for coarse classification of each sector. Here, $SEC_{j,k}$ denotes the j-th longitude and k-th latitude sector.

(1) Statistical Color Features

The statistical color feature vector $f_{j,k}^{b}$ is designed as

(2) $$f_{j,k}^{b}=\left[{\bar{R}}_{j,k},{\bar{G}}_{j,k},{\bar{B}}_{j,k},{\bar{H}}_{j,k},{\bar{S}}_{j,k},{\bar{V}}_{j,k},Std_{j,k}\right]$$

where ${\bar{R}}_{j,k}$, ${\bar{G}}_{j,k}$, and ${\bar{B}}_{j,k}$ are the average values of the Red, Green, and Blue channels for the sector $SEC_{j,k}$, respectively. ${\bar{H}}_{j,k}$, ${\bar{S}}_{j,k}$, and ${\bar{V}}_{j,k}$ are the average values of the Hue, Saturation, and Value channels for $SEC_{j,k}$, respectively. $Std_{j,k}$ represents the standard deviation of the gray values of $SEC_{j,k}$ by the weighted average method.

(2) Gray Variations

The fall-offs of the sealing glass indicate that the fall-off region has less reflection than glass regions. This phenomenon causes the fall-off region in the GIT image to exhibit distinct gray-level characteristics compared with surrounding glass areas. To characterize these gray variations, several feature vectors are designed for each sector, described as following.

Assume that $G_{j,k}^{0}$ is the average gray value of $SEC_{j,k}$, and $G_{j,k}^{L}$, $G_{j,k}^{R}$ and $G_{j,k}^{In}$ are the average gray values of the nearest left, right, and inner neighbor sectors of $SEC_{j,k}$, the local gray variations for $SEC_{j,k}$ are defined as

(3) $$CR_{j,k}^{L}=\frac{G_{j,k}^{0}-G_{j,k}^{L}}{G_{j,k}^{0}}$$

(4) $$CR_{j,k}^{R}=\frac{G_{j,k}^{0}-G_{j,k}^{R}}{G_{j,k}^{0}}$$

(5) $$CR_{j,k}^{In}=\frac{G_{j,k}^{0}-G_{j,k}^{In}}{G_{j,k}^{0}}$$

Equations (3)–(5) indicate that if the absolute value of the local gray variation for $SEC_{j,k}$ is the largest among those of three other local gray variations, the defect may potentially emerge inside the corresponding direction of $SEC_{j,k}$.

The global gray variation $CR_{j,k}^{G}$ for $SEC_{j,k}$ is defined as

(6) $$CR_{j,k}^{G}=\frac{G_{j,k}^{0}-G^{ROI}}{G_{j,k}^{0}}$$

where $G^{ROI}$ is the average gray value of the ROI of the GIT image. As defined above, a larger value of $CR_{j,k}^{G}$ indicates that a higher proportion of pixels in the sector $SEC_{j,k}$ exhibits strong reflective characteristics, which is more likely to occur when the sealing glass fully covers the sector. This is because intact sealing glass tends to produce specular reflections, resulting in higher gray-level responses in the image. In contrast, glass fall-off regions generally exhibit weaker reflection and lower gray-level intensity. Consequently, a smaller value of $CR_{j,k}^{G}$ suggests a higher likelihood that glass fall-off defects emerge within the corresponding sector.

Thus, a gray variation vector $f_{j,k}^{c}$ for the sector $SEC_{j,k}$ is composed as

(7) $$f_{j,k}^{c}=\left[CR_{j,k}^{L},CR_{j,k}^{R},CR_{j,k}^{In},CR_{j,k}^{G}\right]$$

(3) Reflective Features

The sealing glass easily produces the reflection of light, which indicates that the reflection phenomenon is weak in the region with the fall-offs of the sealing glass. To quantitatively characterize the reflective properties of the sector $SEC_{j,k}$, the white ratio $WOR_{j,k}$ and the Canny coefficient $CER_{j,k}$ are designed as two complementary indicators, defined as

(8) $$WOR_{j,k}=1-\frac{\left|SEC_{j,k}^{b}\right|}{\left|SEC_{j,k}\right|}$$

(9) $$SEC_{j,k}^{b}=\{(x,y)\in SEC_{j,k}|Hue(x,y)\in [200,248],Saturation(x,y)\in [43,255],Value(x,y)\in [46,255]\}$$

(10) $$CER_{j,k}=\frac{\left|SEC_{j,k}^{ce}\right|}{\left|SEC_{j,k}\right|}$$

(11) $$SEC_{j,k}^{ce}=\{(x,y)\in SEC_{j,k}|Canny(x,y)=1\}$$

where |S| denotes the cardinality of the set S. Let Canny(x,y) denote the binary edge map obtained by applying the Canny edge detector to the sector image, where edge pixels correspond to strong local intensity gradients. A larger white ratio $WOR_{j,k}$ indicates that a higher proportion of pixels in the sector $SEC_{j,k}$ exhibit higher gray-level responses, while a larger Canny coefficient $CER_{j,k}$ reflects stronger edge responses caused by specular reflection on intact sealing glass surfaces. Therefore, as indicated in Equations (8) and (10), larger values of $WOR_{j,k}$ or $CER_{j,k}$ suggest stronger reflective characteristics of the sector. Then, a reflective feature vector $f_{j,k}^{re}$ for sector $SEC_{j,k}$ is defined as

(12) $$f_{j,k}^{re}=[WOR_{j,k},CER_{j,k}]$$

(4) Statistical Gradient Directions

When some sealing glass falls off, there may be salient edges in the fall-off region; that is, the gray gradient in one direction may be large. Assume G(x,y) as the gray value of any pixel (x,y) in the sector $SEC_{j,k}$, the vertical and horizontal gradients of this pixel are formulated as

(13) $$D_{y}\left(x,y\right)=G\left(x,y+1\right)-G\left(x,y-1\right)$$

(14) $$D_{x}\left(x,y\right)=G\left(x+1,y\right)-G\left(x-1,y\right)$$

Then, the gradient direction of this pixel is

(15) $$\theta \left(x,y\right)=\arctan\frac{D_{y}\left(x,y\right)}{D_{x}\left(x,y\right)}$$

The statistical gradient directions $D_{j,k}^{z}$ (z=1,2,…,9) for the sector $SEC_{j,k}$ can be defined as

(16) $$D_{j,k}^{z}=\frac{\left|SEC_{j,k}^{Dz}\right|}{\left|SEC_{j,k}\right|}$$

(17) $$SEC_{j,k}^{Dz}=\left\{\left(x,y\right)\in SEC_{j,k}|\theta \left(x,y\right)\in \left[40^{\circ }\cdot \left(z-1\right),40^{\circ }\cdot z\right)\right\}$$

Then, a statistical gradient direction vector $f_{j,k}^{d}$ for $SEC_{j,k}$ can be defined as

(18) $$f_{j,k}^{d}=\left[D_{j,k}^{1},\ldots ,D_{j,k}^{9}\right]$$

The larger the variance of $f_{j,k}^{d}$ is, the more probably the defect emerges in the sector.

### 2.4. Fine Classification

**Figure 4:**
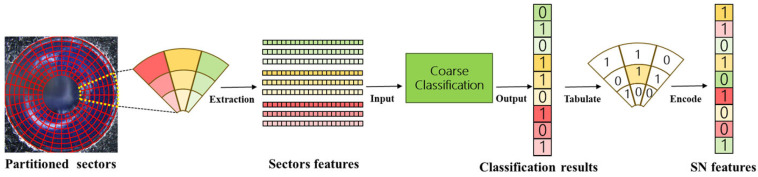
Construction of the SN feature vector. The SN feature vector of each sector is constructed by rearranging their classifications from the upper-left sector in a clockwise order. The numbers 0 and 1 denote the binary coarse classification results, where 1 indicates the presence of glass fall-off and 0 indicates a non-defective sector. Different colors are used to visually distinguish sectors with different classification states and feature components.

Coarse classification alone cannot fully exploit sector neighbor (SN) information and, therefore, fails to capture the local spatial relationships among adjacent sectors. Here, an SN feature vector is designed based on coarse classifications of neighbor sectors, which can be sketched in Figure 4.

When the sector $SEC_{j,k}$ and its eight neighbor sectors are coarsely classified by the GBDT based on the corresponding four categories of sector features described in Section 2.3 coarse classifications for its eight neighbor sectors are rearranged from the upper-left sector in a clockwise order. Thus, an eight-dimensional SN feature vector $SN_{j,k}$ is composed to characterize the relationship between each sector and its neighbor sectors.

Then, a feature vector $F_{j,k}$ can be composed for fine classification by combining the four categories of sector features and the SN feature vector to comprehensively characterize each sector, which is formulated as

(19) $$F_{j,k}=[f_{j,k}^{b},f_{j,k}^{c},f_{j,k}^{re},f_{j,k}^{d},SN_{j,k}{]}^{T}$$

Finally, the feature vector $F_{j,k}$ is input into the GBDT to determine whether glass fall-off defects emerge in the corresponding sector.

### 2.5. Pseudocode of the Proposed Framework

The pseudocode of the proposed coarse-to-fine framework is shown in Algorithm 1.
**Algorithm 1** Proposed Coarse-to-fine Framework**Input**: GIT image **I**, partition parameters (m,n) **Output**: Defect map **D** 1: **H** ← RGB_to_HSV(**I**)      // color space conversion
2: $M_{rough}$ ← BlueMask(**H**)           // rough extraction of sealing-glass region
3: $M_{circle}$ ← MorphologicalRefine($M_{rough}$) // mask refinement and noise suppression
4: (**R**,**r**) ← CircleFitting($M_{circle}$) // estimate outer radius R and inner radius r of the sealing-glass region 5: **ROI** ← RingRegion (**I**, **R**, **r**)     // circular-ring ROI extraction 6: **SEC** ← SectorPartition (**ROI**, **m**, **n**)        // adaptive sector partition of the ROI 
7: **for** each ${SEC}_{j,k}$ ∈ SEC **do**: 
8:          $F_{j,k}$ ← SectorFeatureExtraction(${SEC}_{j,k}$) // extraction of sector-level appearance features
9:          $y_{j,k}$ ← GBDT_coarse ($F_{j,k}$) 10: **end for** 
11: **for** each ${SEC}_{j,k}$ ∈ SEC **do**:
12:        ${SN}_{j,k}$ ← NeighborFeature ($y_{j,k}$)       // construction of sector-neighbor feature
13:        $F_{j,k}^{\prime }$ ← FeatureFusion ($F_{j,k}$, ${SN}_{j,k}$)       // fusion of sector and neighbor information
14:        $D_{j,k}$ ← GBDT_fine ($F_{j,k}^{\prime }$) 15: **end for** 16: **Return D**

## 3. Pixel-Sector Evaluation

In Section 2, we have referred that two partition parameters *m* and *n* correspond to the longitude and latitude directions of the ROI, which determine the number of the partitioned sectors to further influence the inspection. Some commonly used metrics, such as false alarm and missed alarm, can be employed for evaluation to select appropriate partition parameters. In this paper, the Intersection over Union (IoU) is used to evaluate the inspection performance in image level, which is computed between the predicted defect map and the manually annotated ground-truth map inside the extracted ROI. Pixels inside the ROI are classified as either defective (glass fall-offs) or non-defective, while background pixels outside the ROI are excluded from the evaluation. Then, the pixel-level IoU is formulated as

(20) $$IoU=\frac{\left|P\cap G\right|}{\left|P\cup G\right|}$$

where *P* and *G* denote the sets of predicted and ground-truth defective pixels inside the ROI, respectively. Boundary pixels are treated as foreground if they are annotated as defective in the ground truth. Here, the mean IoU is calculated by averaging the IoU scores over all test images under the same experimental setting. To further assess the stability of pixel-level defect localization, the corresponding variance of IoU is additionally reported, formulated as

(21) $$IoU-Var=\frac{1}{N}\sum_{i=1}^{N}{\left({IoU}_{i}-\frac{1}{N}\sum_{i=1}^{N}{IoU}_{i}\right)}^{2}$$

where *N* denotes the total number of test images, and ${IoU}_{i}$ represents the pixel-level IoU value computed for the *i*-th test image between the predicted defect map and the corresponding ground-truth annotation within the ROI.

Since our inspection method extracts sector features for subsequent classification, pixel-level missed alarm (PMA) and pixel-level false alarm (PFA) are utilized for pixel-level evaluation, while sector-level missed alarm (SMA) and sector-level false alarm (SFA) are utilized for sector-level evaluation. These metrics are defined as

(22) $$PMA=\frac{PFN}{PTP+PFN}$$

(23) $$PFA=\frac{PFP}{PTN+PFP}$$

(24) $$SMA=\frac{SFN}{STP+SFN}$$

(25) $$SFA=\frac{SFP}{STN+SFP}$$

where *PFN* and *SFN* denote the numbers of the non-defective pixels and the sectors without defects incorrectly identified as defective, respectively. PFP and SFP denote the number of the defective pixels and the defective sectors incorrectly identified as non-defective, respectively. PTP and STP represent the numbers of the non-defective pixels and the non-defective sectors correctly identified as non-defective, respectively. PTN and STN represent the number of the defective pixels and the defective sectors correctly identified as defective, respectively.

In practice, false alarms and missed alarms are usually contradictory. That is, if the false alarm is controlled to decrease it, the missed alarm usually increases it, and vice versa. Moreover, the increase of the number of partitioned sectors will increase the inspection time. These facts make it challenging to determine appropriate partition parameters by comprehensively evaluating inspection effect (i.e., false alarms and missed alarms) and the inspection efficiency. To this end, a new metric, termed pixel-sector evaluation (PSE), is proposed to comprehensively determine appropriate partition parameters for glass fall-off detection of GITs, which combines pixel-level evaluation and sector-level evaluation with inspection time, formulated as

(26) $$PSE=\frac{\alpha -\lambda \cdot \beta -\delta \cdot \gamma }{t}$$

where t is the inspection time for a GIT image. λ and θ are shrinkage coefficients, which are empirically set to 0.04 and 0.002, respectively. α is a rough parameter to roughly evaluate missed inspection, defined as

(27) $$\alpha =\frac{1}{PMA\cdot SMA}$$

*β* is a balance parameter to comprehensively evaluate the inspection performance for defective samples, defined as

(28) $$\beta =\frac{SFP+STN}{PFP+PTN}\left|\varepsilon \cdot PTN+PFP\right|+\left|\varepsilon \cdot STN+SFP\right|$$

where *SFP* + *STN* and *PFP* + *PTN* denote the total numbers of sector-level and pixel-level defective samples, respectively. *ε* is a penalty factor, which is used to enhance the influence of the defective samples incorrectly identified as non-defective. So, *ε* ∈ (0, 1) and is empirically set to 0.2 in this paper. *γ* is a magnification parameter to evaluate the relationship between the non-defective samples falsely inspected as defective in pixel level and those in sector level, defined as

(29) $$\gamma =\frac{PFP}{SFP}$$

As indicated in Equations (26)–(29), the designed PSE comprehensively takes into account the case that more non-defective pixels are possibly identified as defective if the partition parameters are smaller, and the case that the classifier degrades its performance of classification and the inspection time increases if the partition parameters are larger.

## 4. Experiments and Discussions

### 4.1. Dataset and Experimental Environment

Due to the commercial confidentiality, a total of 43 GITs were provided by a GIT manufacture to the China Electronic Product Reliability and Environmental Testing Research Institute for appearance inspection. The skilled inspector at the China Electronic Product Reliability and Environmental Testing Research Institute carefully observed the sealing glass of all the GITs through a microscope. He manually identified whether and where the sealing glass had fall-offs according to the subtle reflection differences of the sealing glass in the visual field of the microscope. After long-period observations, he identified that 40 ones had glass fall-offs with various degrees, and 3 ones had no glass fall-offs. Then, the images of all the GITs were acquired by a Leica microsystem with a Leica KL300 LED, under the setting of 24-bit bit depth, 10 mm focal length, and aperture set to F/5.6. Each GIT image had the size of 2448 × 1920 pixels. Next, each GIT image was preprocessed by the adaptive sector partition scheme in Section 2.2. Thus, a number of sectors were achieved when the parameters *m* and *n* were determined. The inspection was conducted 10 times. For each inspection, all the sectors of 34 randomly selected images (almost 79% of all images) were utilized to train the proposed framework, and the remaining ones were used for tests. Thus, all the metrics in this section were demonstrated in their average values for 10 inspections.

All the experiments were implemented on a laptop computer with an Intel(R) Core(TM) i5-9300H CPU @ 2.40 GHz, 16 GB of RAM, and a Windows 10 64-bit operating system. The method was programmed by Python 3.10 in PyCharm, using the Scikit-learn toolkit.

### 4.2. Influences of the Partition Parameters

As mentioned in Section 2.3, the two partition parameters *m* and *n* determine the size of each sector, which further influences the inspection effect and inspection efficiency. In this section, we conducted two experiments to evaluate the influence of the partition parameters on the proposed coarse-to-fine machine-learning framework for glass fall-offs detection of GITs. Due to the exponential effect of the partition parameter *m* and the small size of the GIT, a small increase of the partition parameters, especially *m*, results in a greatly large increase of the number of the partitioned sectors. Too many sectors make the inspection significantly time-consuming, which is not acceptable for real practice. Thus, the values of partition parameters were limited within no larger than 5 in the two experiments, that is, *m* = 1, 2, …, 5 and *n* = 1, 2, …, 5.

**Figure 5:**
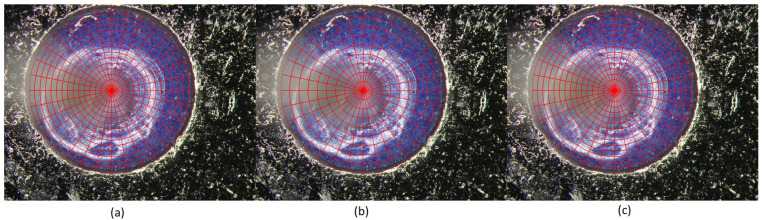
Visualization of the m–n subregion partition strategy. From left to right, the partition parameters are set to (**a**) *m* = 5, *n* = 3; (**b**) *m* = 3, *n* = 3; and (**c**) *m* = 3, *n* = 5.

As shown in Figure 5, the circular-ring region of interest (ROI) is divided into *m* angular sectors and *n* radial rings, resulting in an *m*-*n* subregion grid. Red contours indicate the boundaries of the generated subregions. Increasing *m* leads to finer angular partitioning with narrower sectors, while increasing *n* results in finer radial subdivision with thinner concentric rings.

**Figure 6:**
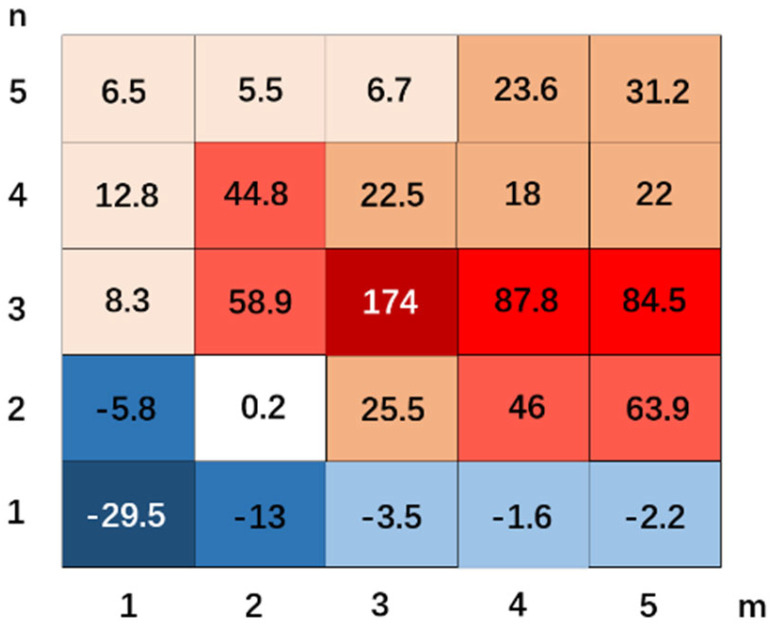
Heatmap achieved by the proposed framework with different partition parameters, which demonstrates the values of the transformation of PSE defined in Equation (30). Warmer colors (red) indicate higher PSE values, while cooler colors (blue) indicate lower or negative values.

To comprehensively evaluate the inspection performance, one experiment was conducted in which PSE, defined in Equation (26), was utilized to evaluate the proposed framework with different partition parameters. For clear visualization of the comprehensive influences of *m* and *n*, the PSE is mathematically transformed with no change in monotony, formulated as

(30) $$\tilde{{PSE}_{nm}}={PSE}_{nm}-\mu +\exp\left({PSE}_{nm}-\mu \right)$$

where ${PSE}_{nm}$ is the PSE value achieved by the proposed framework with the parameters *m* and *n*. μ is the mean of all the PSE values for *M* × *N* proposed frameworks with different parameters (*m* = 1, 2, …, *M* and *n* = 1, 2, …, *N*). Figure 6 illustrates the values of $\tilde{{PSE}_{nm}}$ for the proposed framework with different partition parameters, which is intuitively demonstrated in a heatmap. It is noted that the proposed framework achieves the best inspection performance when the combination of the partition parameters *n*-*m* is 3-3.

To further demonstrate the influences of the partition parameters, a control experiment was conducted. Inspired by the results illustrated in Figure 6, one of partition parameters was controlled to 3 in the control experiment, while the other varied from 1 to 5. As indicated in Table 1, with the increase of *m* and in case of *n* = 3, pixel-level false alarm (PFA) and inspection time increases, while pixel-level missed alarm (PMA) decreases. Simultaneously, sector-level missed alarm (SMA) and sector-level false alarm (SFA) first decrease and then increase. Thus, it is difficult to determine which value is appropriate for m by means of these metrics since a tradeoff emerges between the inspection effects involving false alarms and missed alarms and the inspection efficiency. It is noted that the proposed metrics PSE first increase and then decrease with the increase of m, which is beneficial to determine the appropriate parameter m according to the maximal value of PSE. Also, with the increase of n, and in the case of m = 3, there are similar results illustrated in Table 2. These facts imply that the proposed PSE is beneficial for directly selecting appropriate parameters to achieve the sectors partitioned by a GIT image for inspection. In this paper, this combination (n-m: 3-3) of the partition parameters is utilized for the proposed framework.

### 4.3. Influences of the Classifiers

The proposed framework utilizes the GBDT as the classifier in the stages of coarse classification and fine classification. In this section, we conducted an experiment to discuss the influence of the classifier, in which the combination of n and m is 3-3. Here, for comprehensive evaluation, the proposed PSE is utilized as the metric.

As shown in Table 3, for each classifier, the framework with coarse-to-fine classification achieves better inspection performance than one with coarse classification. That fact validates the coarse-to-fine machine-learning scheme for the proposed framework. Specifically, the framework with the linear discriminant analysis (LDA) and that with the quadratic discriminant analysis (QDA) achieve very poor inspection performance since they are linear classifiers. Classification bias easily emerges when the Gaussian Naïve Bayes (GNB) is utilized for the unbalanced dataset. Thus, the inspection performance of the framework with the GNB is even worse than that with the LDA. Although the SVM has the advantage of non-linear classification, its generalization ability will decrease if the dimension of classification boundaries is greatly high. This will further decrease the inspection ability of the framework with the SVM. Comparatively, the AdaBoost and the GBDT make the framework achieve the two best inspection performances since they are both Boosting methods that can promote weak classifiers to strong classifiers. Compared with the AdaBoost, the GBDT can contribute more to the framework since it can reach the global optimization of the classification model due to its forward stagewise algorithm. So, the GBDT is utilized in the proposed framework to implement the coarse-to-fine machine-learning scheme.

### 4.4. Comparisons with the Existing Inspection Methods

To validate the proposed framework for GIT inspection, it was compared with several related inspection methods, which were the attention model [24], Otsu-based method [25], DCT-based method [26], morphological method [14], and some deep-learning methods [27,28,29]. For fair comparisons, the ROIs were extracted by the proposed adaptive partition scheme from the GIT images for all the inspection methods. Also, several commonly used metrics, such as IoU, F1 measure, PMA, and PFA, were used for evaluation, rather than sector-based metrics proposed in this paper. Table 4 illustrates the statistical comparisons of different inspection methods for glass fall-off detection of GITs.

As shown in Table 4, although the attention model [24] achieves a fairly good false alarm (0.85% PFA), it achieves a very poor missing alarm (93.47% PMA). This is possibly due to its inspection mechanism based on the difference between the adjacent superpixels. However, the sealing region is usually occluded by the pin due to the shooting angle, which results in the superpixels in the occluded region having greatly different characteristics compared with those in the non-occluded sealing region. This great difference will promote the attention model to identify the whole non-occluded region as defective.

Some areas in the sealing region may reflect the lights, which results in them demonstrating higher grayscale values than other areas. Moreover, there are no salient grayscale differences between the fall-off region and the non-fall-off region. Thus, the Otsu-based method [25] achieves the worst pixel-based missing alarm (97.87% PMA), since it is vulnerable to the reflection of the lights. Since it is a histogram-based global thresholding method, its inspection speed can reach 26.89 s/image in our study, which is the fastest among all the non-deep-learning methods involving our method.

The DCT-based method [26] is also influenced by the reflection characteristics of the lights of the sealing glass, since it distinguishes the defects by separating the high-frequency and low-frequency components of the GIT image. Thus, it misidentifies a number of non-defective areas as defective and achieves a poor inspection performance with 83.92% PMA.

The morphological method [14] distinguishes the defects from the connected domains with predefined rules after thresholding, which results in a poor missing alarm with 95.54% PMA. This is because the fall-offs of the sealing glass demonstrate the regions with various appearances and various sizes in the GIT images. Moreover, due to the high computational cost of connected domains, it consumes 513.72 s on average to inspect a GIT image, which is the slowest among all the methods.

The three deep-learning methods have extremely high inspection speeds, ranging from 0.05 s/image to 0.54 s/image, which are significantly faster than the traditional methods. It is noted that these deep-learning–based approaches are not designed as few-shot or small-sample learning methods and, therefore, cannot fundamentally address the limited data scenario considered in this study. Thus, their inspection accuracies are relatively limited for glass fall-off detection. Specifically, RT-DETR and YOLO11 suffer from either high false-alarm rates or high missed-alarm rates, indicating difficulty in distinguishing true defects from reflection-induced artifacts. Although SAM achieves a relatively low false-alarm rate with 0.24% FPA, its IoU and F1 score remain inferior to the proposed framework. These results suggest that, while deep-learning-based methods are advantageous in inspection efficiency, their performances are less stable and less reliable under the reflective and small-sample conditions of GIT inspection. These results highlight the performance degradation of conventional deep-learning models when applied under small-sample and strong-reflection industrial conditions, rather than suggesting their inadequacy in data-rich scenarios.

The proposed framework achieves the best inspection performance with 96.85% IoU, 0.984 F1 measure, 0.55% PFA, and 35.62% PMA at a reasonable inspection speed of 32.18 s per image. Although the proposed method is slower than deep-learning-based approaches, it demonstrates significantly higher accuracy and stability (0.0096% IoU variance), which are critical for reliable glass fall-off inspection in industrial scenarios. This performance advantage can be attributed to three main factors. One is that elaborately designed sector features can reveal the appearance characteristics of each sector. Second, the designed SN feature vector can effectively reveal the local neighbor characteristics of each sector. Third, the proposed coarse-to-fine machine-learning scheme can incorporate the internal and external characteristics of each sector into a framework. It is noted that the pixel-level missed-alarm rate appears relatively high; it mainly arises from partial omission of subtle defect boundaries rather than complete failure to detect glass fall-offs. This relatively high PMA can be attributed to several practical factors, including strong and spatially non-uniform light reflections on intact sealing glass, sector-based representation that prioritizes regional consistency over precise pixel-wise boundary fitting, and the extremely weak contrast of some subtle fall-off regions. These factors may cause fine defect edges or small fragmented fall-offs to be partially missed at the pixel level. From a practical industrial perspective, accurately identifying defective GITs and suppressing false alarms are generally more critical than achieving perfect pixel-wise delineation, making the proposed framework suitable for real-world inspection despite conservative pixel-level boundary estimation.

**Figure 7:**
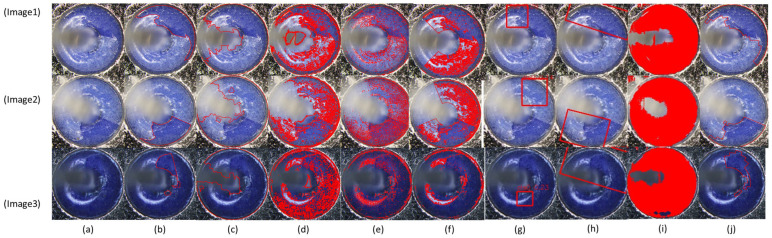
Visualization of inspection results of different methods. (**a**) original image; (**b**) ground truths; (**c**) attention model; (**d**) Otsu-based method; (**e**) DCT-based method; (**f**) Morphological method; (**g**) RT-DETR; (**h**) YOLO11; (**i**) SAM; (**j**) proposed framework. Ground-truth glass fall-offs are manually annotated in yellow. Inspected ones are demonstrated in red. Note that the regions surrounded by red contours refer to inspected glass fall-offs in (**c**,**g**), since they are edge-based inspection methods. Red pixels refer to inspected glass fall-offs in (**d**–**f**) since they are region-based inspection methods.

**Figure 8:**
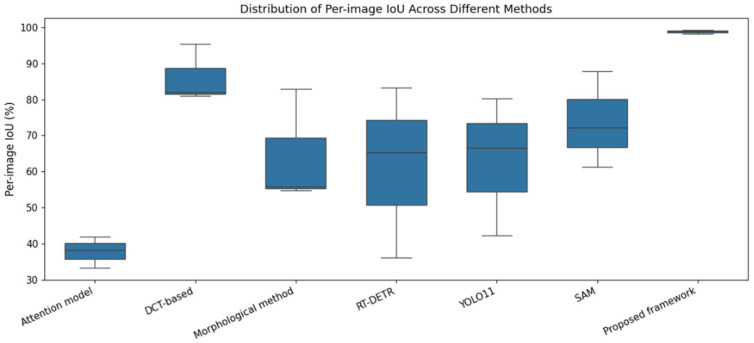
Distribution of per-image IoU values for different inspection methods.

Figure 7 visualizes inspection results of different methods for several GIT samples, whose IoUs are illustrated in Table 5. As indicated in Figure 7, four traditional inspection methods wrongly inspect many regions as fall-off regions. RT-DETR and YOLO11 bound the inspection results due to their bounding-box characteristics. Thus, they either over-inspect or miss-inspect the fall-offs. SAM semantically segments the potential defects from the GIT image, which also wrongly inspects many regions as fall-off regions. Comparatively, our method almost inspects all the fall-offs. More importantly, the distribution of per-image IoU values in Figure 8 demonstrates that the proposed framework achieves not only the highest median IoU but also an extremely compact distribution. The absence of low-IoU outliers indicates that the proposed method maintains consistently accurate defect localization across different GIT samples, effectively reducing the risk of missed fall-off regions. This compact IoU distribution highlights the strong robustness and stability of the proposed framework under varying inspection conditions. These visualization results are consistent with the statistical results.

**Figure 9:**
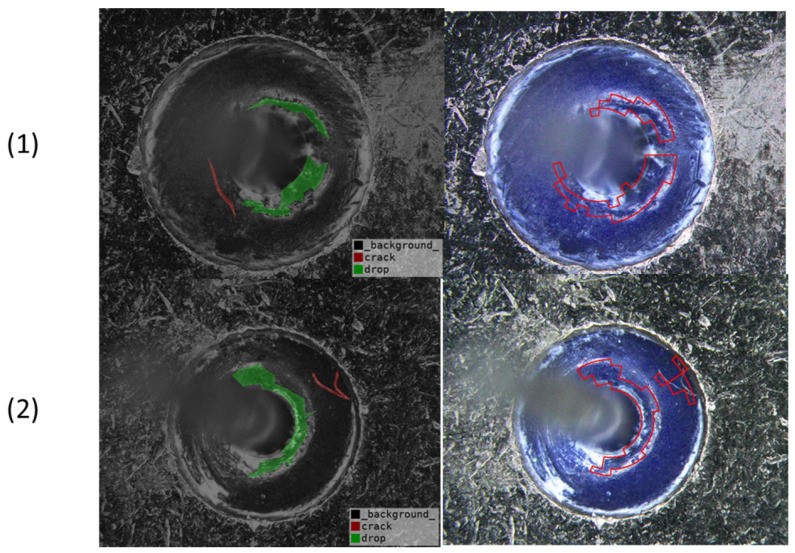
Qualitative results on challenging GIT samples containing crack defects. (1) and (2) correspond to two representative test samples. For each sample, the left and right images show the ground truth and the corresponding inspection result, respectively.

Figure 9 shows representative results on challenging samples containing crack defects. Notably, although the crack regions are generally detected by the proposed framework, the predicted detection contours cannot tightly fit the ground-truth crack boundaries. This is mainly due to the thin, elongated, and irregular morphologies of crack defects, which make precise boundary alignment inherently difficult. Nevertheless, the presence and approximate location of crack defects are correctly identified, indicating that the proposed method remains effective in crack detection.

### 4.5. Evaluation on Another Independent GIT Dataset

**Figure 10:**
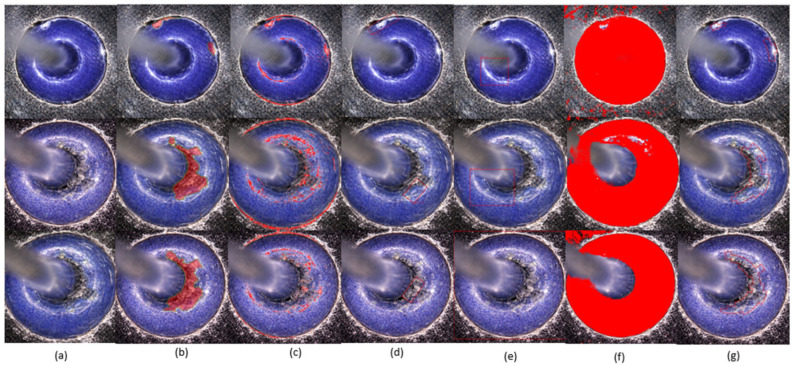
Visualization of inspection results on the independent GIT test dataset (**a**) original images; (**b**) ground truths; (**c**) Morphological method; (**d**) RT-DETR; (**e**) YOLO11; (**f**) SAM; (**g**) proposed framework. The red region represents the inspection region.

To further evaluate the generalization ability of the proposed framework, an additional independent batch of GIT samples was collected for testing. This batch of inspection data was acquired from a different production line but involved GITs of the same product specification as those in the first batch. The inspection conditions, imaging setup, and operating environment remained consistent with the original data collection process. Importantly, this second dataset was not involved in model design, parameter tuning, or feature selection. The qualitative inspection results of different methods on this independent dataset are visualized in Figure 10. For simplicity, we only selected the representative traditional inspection method (i.e., the Morphological method) and the three deep-learning methods for comparisons of the proposed method. All the models trained on the first batch were directly applied to this newly collected dataset without any retraining or adaptation, enabling objective assessments of generalization capabilities.

As indicated in Table 6, the proposed framework consistently achieves the best inspection performance in terms of IoU, F1 score, false-alarm rate, and missed-alarm rate while maintaining reasonable inspection efficiency. In contrast, the performance of deep-learning-based methods degrades significantly on the new data, which can be attributed to strong reflection interference and limited generalizations under small-sample industrial conditions. These results demonstrate that the proposed coarse-to-fine framework exhibits a superior generalization capability when applied to unseen industrial GIT samples, further validating its practical applicability.

## 5. Conclusions

A coarse-to-fine machine-learning framework is proposed in this study for automatic inspection of glass fall-offs in glass-insulated terminals (GITs) under complex reflective imaging conditions. By exploiting the circular-ring geometric prior of GITs, the framework integrates adaptive sector partitioning, handcrafted sector features, and sector neighbor (SN) information into a unified coarse-to-fine classification scheme, enabling robust defect localization under strong reflection interference and limited defective samples.

Quantitative comparisons with several traditional inspection methods and representative deep-learning-based approaches demonstrate that the proposed method achieves superior inspection accuracy and stability, with an IoU of 96.85 ± 0.0096%, an F1 measure of 0.984, a low false-alarm rate of 0.55%, and a competitive missed-alarm performance. Moreover, per-image IoU analysis and the introduction of IoU variance further verify the consistency of the inspection results. Additional experiments on an independent GIT dataset collected from a different production line confirm the strong generalization capability of the proposed framework under real industrial conditions.

Although the proposed framework is slower than deep-learning-based methods, its inspection speed remains practical for industrial applications where accuracy and reliability are prioritized over extreme real-time performance. Future work will focus on improving inspection efficiency without sacrificing accuracy, such as optimizing sector partition strategies, reducing feature redundancy through feature selection, and accelerating feature extraction and classification via parallel processing. These improvements are expected to further enhance the applicability of the proposed framework for large-scale industrial deployment.

## Figures and Tables

**Table 1:** Inspection performance achieved by the proposed framework with different *m*.

n-m	PMA (%)	PFA (%)	SMA (%)	SFA (%)	Time (s/Image)	PSE
3-1	50.49	**0.53**	1.98	0.29	**31.24**	−0.38
3-2	41.97	0.55	**1.69**	**0.27**	31.80	1.76
3-3	35.62	0.55	1.89	0.30	32.18	**2.65**
3-4	29.51	0.62	3.16	0.34	33.16	2.18
3-5	**26.34**	0.66	3.60	0.51	34.79	2.02

**Table 2:** Inspection performance achieved by the proposed framework with different *n*.

n-m	PMA (%)	PFA (%)	SMA (%)	SFA (%)	Time (s/Image)	PSE
1-3	58.81	0.67	1.5	0.39	**30.42**	5.75
2-3	46.35	**0.53**	**1.1**	**0.30**	30.90	0.87
3-3	35.62	0.55	1.9	**0.30**	32.18	**2.65**
4-3	26.69	0.79	4.9	0.71	34.77	0.73
5-3	**17.23**	1.55	13.65	1.33	39.59	0.01

**Table 3:** Comparisons of different Classifiers.

Classifiers	Coarse Classification	Fine Classification
GBDT	**−0.85**	**2.65**
GNB	−5.2	−4.87
LDA	−4.79	−4.28
QDA	−8.85	−5.19
SVM	−3.08	−1.96
AdaBoost	−0.88	1.23

**Table 4:** Comparisons of different inspection methods for GITs.

Methods	IoU (%)	F1	PFA (%)	PMA (%)	Time (s/Image)
Attention model [24]	35.09 ± 4.11	0.519	0.85	93.47	88.08
Otsu-based [25]	60.04 ± 8.74	0.750	5.82	97.87	26.89
DCT-based [26]	89.70 ± 0.040	0.945	4.28	83.92	30.27
Morphological method [14]	80.68 ± 4.26	0.893	4.17	95.54	513.72
RT-DETR [27]	65.62 ± 3.22	0.800	80.77	50.00	0.075
YOLO11 [28]	72.26 ± 2.77	0.375	85.71	90.00	0.051
SAM [29]	71.76 ± 1.84	0.836	**0.24**	**7.76**	**0.54**
Proposed framework	**96.85 ± 0.0096**	**0.984**	0.55	35.62	32.18

**Table 5:** IoUs (%) for visual inspection results shown in Figure 7.

Methods	Image 1	Image 2	Image 3
Attention model [24]	38.19	33.29	42.03
Otsu-based [25]	59.33	65.78	51.09
DCT-based [26]	95.36	80.94	82.11
Morphological method [14]	55.83	54.75	82.89
RT-DETR [27]	83.34	36.22	65.29
YOLO11 [28]	80.27	66.58	42.29
SAM [29]	87.90	72.16	61.27
Proposed framework	**98.28**	**98.97**	**99.26**

**Table 6:** Comparisons on an independent GIT test dataset.

Methods	IoU (%)	F1	PFA (%)	PMA (%)	Time (s/Image)
Morphological method [14]	75.28 ± 2.14	0.8590	6.33	89.70	378.9
RT-DETR [27]	45.27 ± 0.01	0.3333	92.86	91.18	0.0320
YOLO11 [28]	68.25 ± 0.96	0.3750	90.00	97.06	**0.0205**
SAM [29]	44.96 ± 0.043	0.6204	4.53	46.03	0.5675
Proposed framework	**94.48 ± 0.029**	**0.9716**	**0.16**	**0.38**	34.95

## Data Availability

The datasets used and/or analyzed during the current study are available from the corresponding author upon reasonable request.

## Abbreviations

AOI	Automatic Optical Inspection
Canny	Canny edge detector
DCGAN	Deep Convolutional Generative Adversarial Network
DCT	Discrete Cosine Transform
FPA	False Positive Area
GBDT	Gradient Boosting Decision Tree
GIT	Glass Insulated Terminal
GNB	Gaussian Naïve Bayes
HSV	Hue, Saturation, and Value
IoU	Intersection over Union
LDA	Linear Discriminant Analysis
PFA	Pixel-level False Alarm
PMA	Pixel-level Missed Alarm
PSE	Pixel-Sector Evaluation
QC	Quality Control
QDA	Quadratic Discriminant Analysis
ROI	Region of Interest
RT-DETR	Real-Time Detection Transformer
SAM	Segment Anything Model
SN	Sector Neighbor
SFA	Sector-level False Alarm
SMA	Sector-level Missed Alarm
SVM	Support Vector Machine
YOLO	You Only Look Once
